# Genetic Control of Startle Behavior in Medaka Fish

**DOI:** 10.1371/journal.pone.0112527

**Published:** 2014-11-13

**Authors:** Satomi Tsuboko, Tetsuaki Kimura, Minori Shinya, Yuji Suehiro, Teruhiro Okuyama, Atsuko Shimada, Hiroyuki Takeda, Kiyoshi Naruse, Takeo Kubo, Hideaki Takeuchi

**Affiliations:** 1 Department of Biological Sciences, Graduate School of Science, The University of Tokyo, Bunkyo, Tokyo, Japan; 2 Laboratory of Bioresources, National Institute for Basic Biology, Okazaki, Aichi, Japan; 3 National Institute for Basic Biology Center of the Interuniversity Bio-Backup Project, National Institute for Basic Biology, Okazaki, Aichi, Japan; 4 Model Fish Genomics Resource, Genetic Strains Research Center, National Institute of Genetics, Mishima, Shizuoka, Japan; 5 Department of Biology, Keio University, Yokohama, Kanagawa, Japan; 6 Department of Physiology, Tokyo Women's Medical University School of Medicine, Shinjuku, Tokyo, Japan; National Institutes of Health/NICHD, United States of America

## Abstract

Genetic polymorphisms are thought to generate intraspecific behavioral diversities, both within and among populations. The mechanisms underlying genetic control of behavioral properties, however, remain unclear in wild-type vertebrates, including humans. To explore this issue, we used diverse inbred strains of medaka fish (*Oryzias latipes*) established from the same and different local populations. Medaka exhibit a startle response to a visual stimulus (extinction of illumination) by rapidly bending their bodies (C-start) 20-ms after the stimulus presentation. We measured the rates of the response to repeated stimuli (1-s interval, 40 times) among four inbred strains, HNI-I, HNI-II, HO5, and Hd-rR-II1, and quantified two properties of the startle response: sensitivity (response rate to the first stimulus) and attenuation of the response probability with repeated stimulus presentation. Among the four strains, the greatest differences in these properties were detected between HNI-II and Hd-rR-II1. HNI-II exhibited high sensitivity (approximately 80%) and no attenuation, while Hd-rR-II1 exhibited low sensitivity (approximately 50%) and almost complete attenuation after only five stimulus presentations. Our findings suggested behavioral diversity of the startle response within a local population as well as among different populations. Linkage analysis with F2 progeny between HNI-II and Hd-rR-II1 detected quantitative trait loci (QTL) highly related to attenuation, but not to sensitivity, with a maximum logarithm of odds score of 11.82 on linkage group 16. The three genotypes (homozygous for HNI-II and Hd-rR-II1 alleles, and heterozygous) at the marker nearest the QTL correlated with attenuation. Our findings are the first to suggest that a single genomic region might be sufficient to generate individual differences in startle behavior between wild-type strains. Further identification of genetic polymorphisms that define the behavioral trait will contribute to our understanding of the neural mechanisms underlying behavioral diversity, allowing us to investigate the adaptive significance of intraspecific behavioral polymorphisms of the startle response.

## Introduction

Many animals, including humans [1], exhibit intraspecific behavioral diversities [2], [3], [4], [5], [6], which are generated not only by learning and development, but also by genetic factors [2], [3]. Individual differences in behavioral traits are thought to be an important factor influencing fitness [7] and are suggested to be under natural selection [2]. In addition, intraspecific behavioral diversities in some animal groups may contribute to the emergence of social organization [8], [9]. Thus, individual differences in behavioral characteristics have attracted the interest of many researchers in the field of evolutionary ecology [1], [2], [4], [5]. Why individual differences are maintained in natural populations and how genetic polymorphisms generate behavioral diversities, however, remain unresolved.

To explore this issue, we used medaka fish (*Oryzias latipes*). Over 10 inbred strains of medaka generated from over 20 generations of sister-brother mating have been established from various origins, including wild and pet populations [10], [11], [12], [13], [14]. Importantly, the availability of a dense genetic linkage map [15], [16], [17], [18] and large-scale genomic data [19], [20], [21], [22] allows us to identify the quantitative trait loci (QTL) for complex traits and to compare genetic polymorphisms in candidate genomic regions among medaka inbred strains. Comparative analysis of the genomes of medaka inbred strains revealed a genome-wide single nucleotide polymorphism rate between the two inbred strains (HNI-II and Hd-rR-II1) of 3.42%, the highest single nucleotide polymorphism rate observed to date in any vertebrate species [19], [20]. In the process of inbreeding, the genetic variation within the species led to differences among strains. Because each inbred strain is genetically homogeneous with its unique genotype, the genetic background itself is thought to define strain-specific traits [15], [23]. Medaka are thus considered an ideal animal model for the search of genetic factors that underlie complex traits [15].

To date, there is only one report describing differences in behavioral traits among medaka inbred strains; some inbred strains tend to be attached to humans, while other inbred strains tend to escape from humans [13], responses that may be mediated by a visual stimulus. The difference in behavioral traits, however, has not been quantitatively analyzed based on paradigmatic experiments. In the present study, we quantitatively compared the visual response among medaka inbred strains and performed a genome-wide QTL analysis.

## Results

### Behavioral paradigm of visually-evoked startle response

**Figure 1:**
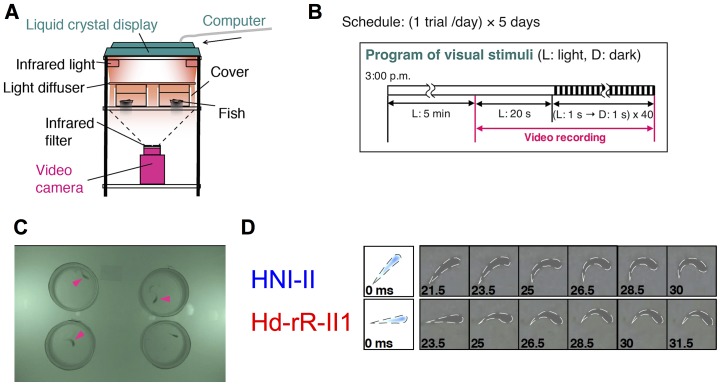
Experimental Design and Observed Startle Response. (A) Experimental apparatus with infrared imaging system. (B) Experimental program. (C) Images from infrared recording. Three individuals (top-left, top-right, and bottom-left, indicated by magenta arrowheads) exhibit C-start. (D) C-start of medaka. Fish bend their body in a “C” shape after a ∼20-ms latency.

We established a behavioral paradigm to quantify visually-evoked behavioral characteristics of medaka using a liquid crystal display (Figure 1A). Russel (1967) demonstrated that a shadow stimulus induces a startle response in guppies [24]. We found that extinction of illumination (color transition from white to black on the entire screen of the liquid crystal display) robustly elicited a startle response in medaka fish. At approximately 20-ms after the stimulus presentation, medaka exhibited rapid and transient body bending (Figure 1C and 1D). This startle response resembled the “C-start”, which is broadly observed in the escape behaviors of teleost fish [25]. Medaka fish exhibited a response to the extinction of illumination (light-to-dark stimulus), but no apparent response to dark-to-light stimulus (Movie S1 and S2). We tested four inbred strains from the same and different populations (Table 1, [11], [12], [13], [14]), and detected no prominent difference in either the latency of the response or the motion kinematics.

**Table 1:** Inbred Strains and Their Origins.

Strain	Local Population	Origin
HNI-I	Northern Japan	Wild population in Niigata city, Niigata Prefecture, Japan
HNI-II	Northern Japan	Same wild population as HNI-I
HO5	Southern Japan	Commercial stock purchased in Chiba prefecture, Japan
Hd-rR-II1	Southern Japan	Commercial stock purchased in Yatomi, Aichi prefecture, Japan

### Differences in startle response properties among medaka inbred strains

**Figure 2:**
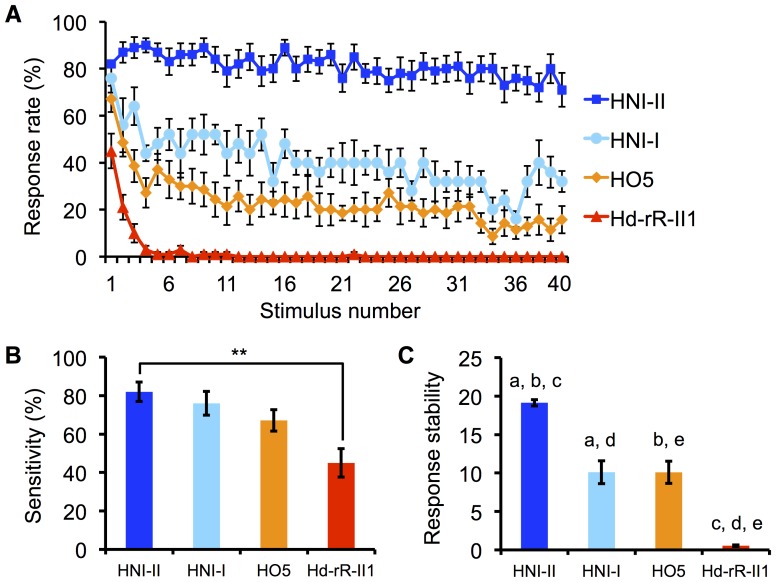
Comparison of Startle Response Properties among Inbred Strains. HNI-II (n = 20), HNI-I (n = 6), HO5 (n = 14), Hd-rR-II1 (n = 20). (A) Transition of response probability. Bars represent SEM. (B) Sensitivity of four inbred strains. Bars represent SEM. ** p<0.01 by Scheffe's F test. (C) Response stability index of four inbred strains. Bars represent SEM. a, b, c, d, and e indicate p<0.01 by Scheffe's F test.

To investigate changes in the startle response to repeated eliciting stimuli, we used blinking stimuli, where the entire screen flickered 40 times at 1-s intervals (Figure 1B). We examined whether fish exhibited the startle response to each eliciting stimulus based on recorded movies (Figure 1A and 1C). This behavioral test was performed in the same fish everyday for five consecutive days and the startle response rate of each fish was calculated for every stimulus (Figure 2A). Among the four strains, the most prominent differences were detected between HNI-II and Hd-rR-II1 in two features, although neither strain exhibited a significantly decreased maximum velocity during the response (i.e. response magnitude) and both continued to respond (one-way ANOVA; HNI-II: F (39, 685)  = 1.14, p>0.05; Hd-rR-II1: F (3, 28)  = 2.11, p>0.05; Figure S1A). HNI-II demonstrated the highest sensitivity and no attenuation of response probability, and Hd-rR-II1 demonstrated the lowest sensitivity and almost complete attenuation of response probability within five repeated stimulus presentations. To quantitatively compare the response properties, we defined two indexes. The “sensitivity index” was defined as the probability of a response to the first eliciting stimulus on each of the 5 days. The “response stability index” was defined as the number of eliciting stimuli needed before an integrated response reached half the total amount (see Materials and Methods), which negatively correlates with the degree of attenuation. No effect of day on either of the indices was detected (two-way repeated measures ANOVA; sensitivity: F (4, 280)  = 0.31, p>0.05; response stability index: F (4, 280)  = 1.03, p>0.05; Figure S2). The sensitivity index was significantly different between HNI-II and Hd-rR-II1 (Scheffe's F test, p<0.01; Figure 2B and S3A) among the four inbred strain, while the response stability index was significantly different among several combinations (Figure 2C and S3B). HNI-I and HNI-II both originated from the same local-wild population or a “deme” (Niigata stock), belonging to the Northern Japan population [13], [14], [20], while Hd-rR-II1 and HO5 originated from different stocks in the Southern Japan population [13], [14], [20].

### Attenuation of the startle response probability with highly frequent stimuli in HNI-II

**Figure 3:**
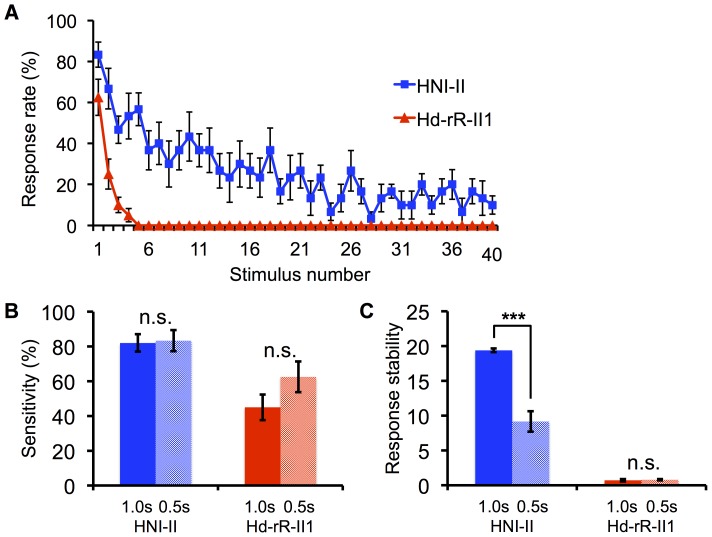
Comparison of Startle Response Properties between Different Stimulus Interval Conditions. HNI-II (n = 6), Hd-rR-II1 (n = 8). (A) Transition of response probability to frequent (0.5-s interval) stimulus presentation. (B) Sensitivity in different stimulus interval conditions. Bars represent SEM. In each inbred strain, no significant difference was detected between the 1.0-s and 0.5-s interval conditions, based on one-way ANOVA. (C) Response stability index of two inbred strains. Bars represent SEM. ***p<0.001 by one-way ANOVA.

HNI-II exhibited no attenuation in response probability to repeated stimulus presentations, suggesting that some mutations in the HNI-II inbred strain resulted in a loss of the ability to exhibit startle response attenuation. Therefore, to examine whether HNI-II could exhibit startle response attenuation, we presented visual stimuli with a shorter interval, i.e., the display flickered 40 times at 0.5-s intervals. Under these conditions, HNI-II exhibited an attenuated startle response and the response stability index was significantly lower than that when stimuli were presented at 1.0-s intervals (one-way ANOVA; F (1, 24)  = 83.32, p<0.001; HNI-II, n = 6; Hd-rR-II1, n = 8; Figure 3C). This finding indicated that HNI-II exhibits attenuated startle response probability to repeated stimulus presentations. The index in Hd-rR-II1, however, did not differ significantly between the two stimulus presentation conditions (one-way ANOVA; F (1, 26)  = 1.68, p>0.05; Figure 3C), suggesting that the response stability index of Hd-rR-II1 reached the lowest plateau with presentation at 1.0-s intervals. There was no significant difference in sensitivity in either HNI-II or Hd-rR-II1 (one-way ANOVA; HNI-II: F (1, 24)  = 0.02 p>0.05; Hd-rR-II1: F (1, 26)  = 1.82, p>0.05; Figure 3B). Thus, we demonstrated that HNI-II does not have defective plasticity of the visual response.

### Identification of QTL highly related to startle response attenuation

**Figure 4:**
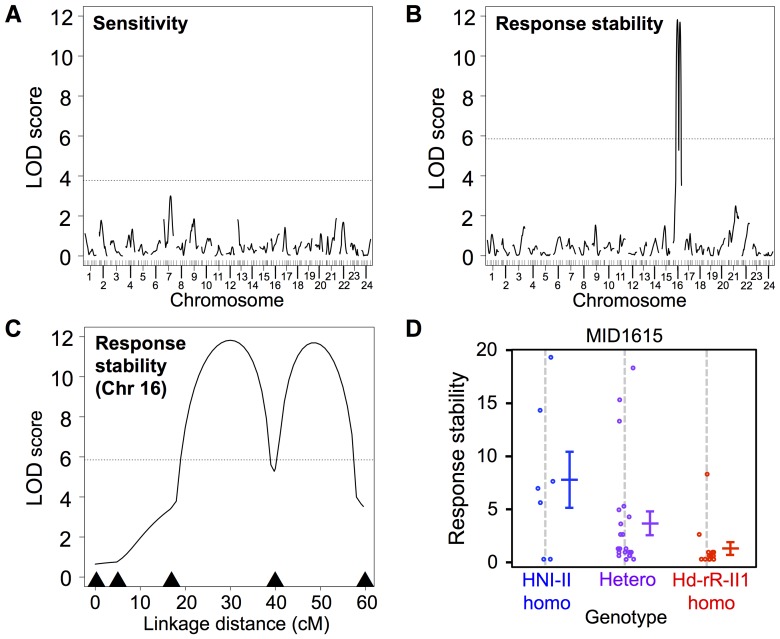
Quantitative Trait Analysis with F2 between HNI-II and Hd-rR-II1. In A, B, and C, dashed lines indicate the thresholds (p = 0.05). (A) QTL analysis on sensitivity. No significant QTL was detected. (n = 87) (B) QTL analysis on response stability index. Significant QTL was detected on chromosome 16. (n = 78) (C) QTL analysis on response stability index. The result of chromosome 16 is shown. Black arrowheads indicate marker positions. (D) Genotype-phenotype relationship at a polymorphic marker located at 39.75-cM on linkage group 16, corresponding to chromosome 16.

To search for candidate genetic regions defining the behavioral diversity of startle response attenuation, we performed a QTL analysis using HNI-II and Hd-rR-II1. We selected the two strains for their most prominent behavioral difference (Figure 2B and 2C), abundance of polymorphic markers [15], [17], [26], and availability of genome sequence information [20] for further investigations. For QTL analysis, we crossed HNI-II and Hd-rR-II1 for two generations and obtained ∼100 F2 individuals. The sensitivity and response stability indices were measured in each individual under the 1-s interval stimulus presentation condition. After the behavioral tests, each individual was genotyped with 147 polymorphic markers on the genome, indicated as black bars in Figure 4A and 4B, and black arrowheads in Figure 4C, with a marker interval length of ∼10-cM.

Analysis of the response stability index detected revealed significant QTL with a maximum logarithm of odds (LOD) score of 11.82 located on linkage group 16, explaining 26.7% of the variance (n = 78, p<0.01; Figure 4C, 4D and Table 2). The response stability index of F1 between HNI-II and Hd-rR-II1 was similar to that of Hd-rR-II1 (Figure S4C), suggesting that recessive genetic factors may have a major effect on the behavioral traits of HNI-II.

**Table 2:** Location and Effect of the QTL Detected in the Analysis with the Whole F2 Population.

Trait	LG	Estimated QTL (cM)	LOD score	Nearest marker	Marker location (cM)	LOD score	p Value	PVE	HNI-II homo	Hetero	Hd-rR-II1 homo
Sensitivity	-	-	-	-	-	-	-	-	-	-	-
Response stability index	16	30	11.82	MID1615	39.75	5.26	p<0.001	26.7	8.9±1.1	3.1±0.7	1.4±1.0

PVE, percent variance explained; HNI-II homo, homozygous for HNI-II alleles; Hetero, heterozygous, Hd-rR-II1 homo, homozygous for Hd-rR-II1 alleles.

PVE, percent variance explained; HNI-II homo, homozygous for HNI-II alleles; Hetero, heterozygous, Hd-rR-II1 homo, homozygous for Hd-rR-II1 alleles.

Analysis of the sensitivity index with the entire F2 population revealed no significant QTL (n = 87, p>0.05; Figure 4A). In the previous F1 generation, the sensitivity index was lower in progeny between Hd-rR-II1 females and HNI-II males (F1-a) than that of HNI-II, while the index of progeny obtained from the reciprocal cross (F1-b, from HNI-II females and Hd-rR-II1 males) was not significantly lower than that of HNI-II (Figure S4B). In the original four inbred strains we used, the sensitivity index of females was significantly higher than that of males (two-way ANOVA; sex: F (1,52)  = 9.61, p<0.01; Figure S5A), while there was no significant difference in the response stability index between sexes (two-way ANOVA; sex: F (1,52)  = 2.63, p = 0.11; Figure S5B). Thus, the failure to detect a QTL for the sensitivity index might be due to a sexual dimorphism of this behavioral trait. Analysis discriminating F2 populations from different crosses suggested the presence of a QTL that was specifically detected with the offspring of F1-a (n = 29, p<0.05; Figure S6A), not with that of F1-b (n = 58, p>0.05; Figure S6B), which might support the hypothesis. This QTL was not on linkage group 1, corresponding to the sex chromosome of medaka [27].

## Discussion

The findings of the present study demonstrated a significant difference in the startle response pattern among medaka inbred strains. The startle response is a reflexive reaction to potentially stressful environmental stimulation that is observed in most, if not all, animal species [28]. Extinction of illumination robustly induces the startle response (C-start) in medaka fish, suggesting that the visual stimulus mimics the sudden appearance of a natural predator above the water [24], [29]. In rodents, rats exhibit a startle response to an acoustic stimulus, and there is a significant difference among rodent strains in both the mean amplitude of the startle response and startle response attenuation to repeated startle stimulus presentations (15-s interval) [30]. The genetic polymorphism that determines the difference in the startle response among rodent wild strains based on QTL analysis has not been clarified [31], [32], [33]. In the present medaka study, QTL analysis suggested that a single 30-cM genomic region (18 Mbp) mainly defines the difference in startle response attenuation. This genomic region contains approximately 500 predicted genes. Further generation of congenic lines will allow us to narrow candidate genomic regions to a restricted region containing ∼100 predicted genes and to examine epistatic effects between other loci. The applicability of recently developed advanced molecular genetic techniques, such as transgenic and genomic editing [34], [35], [36], [37], to medaka will allow for the identification of additional genetic polymorphisms [38]. The identification of genetic factors underlying the diversities of the startle response will allow us to investigate the ultimate causes of individual behavioral differences in medaka fish.

Medaka are thought to be a model organism for comparative population genetics [11], [39], [40], [41]. The availability of various local-wild medaka populations further allows for investigations of the adaptive meaning of behavioral diversities defined by genetic polymorphisms among populations and estimations of the evolutionary trajectory of behavioral traits [11], [39], [40], [41]. Medaka fish exhibit various kinds of social behaviors, such as female mating preference [42], schooling [43], [44], visually-guided group-foraging [45], and behavioral paradigms to quantify these social behaviors have been already established, which will allow us to examine how the behavioral diversities within animal groups are involved in the emergence of social organization [8] and collective decision-making [38]. Our results demonstrated differences in the startle response between HNI-I and HNI-II, which both originated from the same local-wild population. Further behavioral and genetic characterizations of individuals in wild populations will help us to determine whether the behavioral differences reflect genetically-affected behavioral diversity within a local-wild population. Our study reveals the future potential of medaka as a new model animal for behavioral genetics as well as evolutionary behavioral ecology.

## Materials and Methods

### Ethics statement

The work in this paper was conducted using protocols approved by the Animal Care and Use Committee of the University of Tokyo (permit number: 12–07). All surgery was performed under cold anesthesia, and all efforts were made to minimize suffering.

### Animals

Adult medaka (>3 months after hatch, 2.5–4 cm of length) were used for behavioral tests and genotyping. Four medaka inbred strains, HNI-I, HNI-II, Hd-rR-II1, and HO5 [11], [12], [13], [14], were used for inter-strain behavioral comparison. These strains have been established and maintained by over 20 generations of full sister-brother mating. All of these strains were provided by the National BioResource Project Medaka. Two F1 hybrids, F1-a (female Hd-rR-II1 x male HNI-II) and F1-b (female HNI-II x male Hd-rR-II1), were used for the experiment shown in Figure S4. The two F2 hybrids used for the QTL analysis, F2-a and F2-b, were obtained from sibling mating within F1-a and F1-b, respectively. Water temperature was maintained at ∼28°C. The light: dark ratio was 14 h∶10 h, with the lights on from 08:00 to 22:00. Fish were maintained in plastic aquariums (12 cm×13 cm×19 cm). Each aquarium was separated by a transparent partition into two ID-tagged compartments, each of which contained two fish. Fish were fed brine shrimp (3 - 4 times daily). Rearing water was circulated among the aquariums, and the loss was replaced with dechlorinated tap water.

### Behavioral tests

The behavioral tests were performed over 5 days. On each experimental day, fish were scooped from the aquariums at ∼12:00, and placed into ID-tagged plastic cups with rearing water (one pair of fish per cup) for transportation to the observation room. Fish were kept in the observation room for ∼3 hours for acclimation. For behavioral observation, a shelf with three transparent glass boards was used (Figure 1A). At ∼15:00, each individual fish was placed in a tall Petri dish (8 cm in diameter, filled with water to a 2–3 cm depth, and covered on the side surface with white paper) on the middle shelf with a 4-cm-long plastic plate as a spatial scale. Up to four fish were tested at the same time, and the other fish were kept under an opaque cover during the trial. After the fish were set on the middle board, the light in the experiment room was turned off. Visual stimuli were presented with a liquid-crystal display (TSA4634JT, Iiyama, Tokyo, Japan. Response time: 72 ms) set on the upper board of the shelf. Fish were first presented with the light stimulus for 5 min for acclimation, and then for 20 s for start-up of the video recording. After the video recording was started, fish were shown a stimulus movie (Figure 1B). Video recording was stopped after the movie finished. Between trials, the light of the room was turned on to change fish. After all trials were complete, the fish were transported back to the rearing room.

### Visual stimuli

In the trials, fish were presented a light-dark stimulus movie in which 40 light periods and 40 dark periods appeared alternately. In the experiments shown in Figure 2, 4, S1, S2, S3, S4, S5 and S6, the lengths of the light periods (interval of dark stimuli) and dark periods were each set to be 1.0-s long. In the experiment shown as “0.5 s” in Figure 3, the dark stimulus was presented every 0.5-s for a period of 1.0-s.

### Video recording

The time-lapse image of C-start shown in Figure 1D was recorded at 600 frames per second with a digital camera (EXILIM EX-F1, CASIO, Japan). In the experiments shown in Figure 2, 3, 4, S1, S2, S3, S4, S5 and S6, the infrared imaging (“night-shot”) mode of a video camera (HDR-HC3, SONY, Japan) was used to observe the behavior of the fish under consecutive light-to-dark transitions. Infrared LED lights (LB12-WP01, Ebisu-denshi, Osaka, Japan, with a peak wavelength of 850 nm that is out of the estimated visible wavelength range of medaka [46]), were used for infrared lighting. To illuminate the viewing field uniformly, an opalescent acrylic board (3 mm thick) was placed on tall Petri dishes as a light diffuser. The movement of the fish was recorded from underneath at 30 frames per second with the video camera. To cut off excess visible light from the recorded movies, an infrared filter (IR 78, Fuji film, Japan), which cuts off light with a wavelength <780 nm, was set on the lens of the video camera. A pinhole was created at the center of the filter, through which the light-dark changes of the display were recorded (Movie S2).

### Quantification of response properties

The startle response was manually scored on the video recordings (30 frames per second), as a quick tail flip with C-shaped body bend starting within three recording frames after an onset of light-to-dark transition, with about>0.5 cm of body moving distance between two adjacent recording frames (corresponding to >15 cm/s in velocity). The “sensitivity” was calculated as response probability to the first dark period. The “response stability index” was calculated as the number of dark periods needed before an integrated amount of responses reached half the total amount of response. In the analysis of sensitivity, a mean value of 5 days was used as a characteristic index value for each individual. In the analysis of the response stability index, a mean value of 3 days, not including the highest and lowest values over the 5 days, was used for each individual.

### Quantification of maximum velocity during a response

The maximum velocity during each response was calculated from fish velocities and time sequences of light-dark changes according to the following procedure using ImageJ (http://imagej.nih.gov/ij/). First, the recorded movies were imported to ImageJ (Ver.10.2) using QuickTime (Ver. 7.6.4) and each frame of the movies was converted to an 8-bit monochrome image. To calculate the fish velocities, the area inside of the tall Petri dish was defined as the regions of interests. The brightness threshold was set to distinguish the fish from the background, and a spatial scale was calibrated using a 4-cm-long plastic plate placed near the tall Petri dish. The positions of the fish were measured using the Particle Analyzer algorithm of ImageJ and the velocities of the fish were calculated at each frame. To track light-dark changes over time, the “Stacks” function of ImageJ was used. The small circular area at the center of the image, representing the light-dark changes recorded through the pinhole of the infrared filter, was selected as a region of interest (see Movie S2). A time sequence of brightness in the region was obtained by the “Plot Z-axis Profile” command, and the onset of each light-to-dark change was defined as <−1 of transition in brightness from a previous recording frame (in typical cases, mean brightness values in the region were ∼85 in light states, and <80 in dark states). Based on the sequential values, the maximum velocity during each response was defined as the highest velocity in three recording frames after a light-to-dark transition.

### Genotyping

For DNA extraction, fish were euthanized by submersion in ice water (4°C or less) until cessation of gill movement and heartbeat, and then decapitated with a sharp blade. Genomic DNA was extracted using a PI-50 automatic DNA isolation system (Kurabo Industries Ltd., Osaka, Japan) from the decapitated and gutted medaka bodies fixed in 100% ethanol. We used 147 polymerase chain reaction product length polymorphism primers, which were described previously [26]. Polymerase chain reactions and polymorphic analysis were performed as described previously [16], [26].

### QTL analysis

We used a total of 117 F2 individuals for 5 days of behavioral tests. Behavioral tests were performed in 10 divided batches. In each batch, 2 HNI-II (positive control), 2 Hd-rR-II1 (negative control), and 11 or 12 F2 individuals were used. In preliminary experiments, some batches exhibited a low response rate among all individuals, including HNI-II. Therefore, we used the response rate of HNI-II as a criterion for excluding batches with generally low response rates from a dataset for further analysis. We eliminated two batches (23 individuals) in which HNI-II exhibiting <70% of the mean response rates throughout the 40 stimulus presentations. We also eliminated four individuals that died after the behavioral tests, and three individuals for which the movie recordings were problematic. As a result, a total of 87 F2 individuals (offspring of F1-a: n = 29, offspring of F1-b: n = 58) were used for further QTL analysis. In the analysis of the response stability index, nine individuals with no response were additionally eliminated from the dataset, because we could not define the index in such cases. QTL analysis was performed using R/qtl [47]. Nonparametric interval mapping was performed with a “scanone” function, using the argument “model  =  np”. The LOD threshold for interval mapping was determined by 1000 iterated permutations. The genome-wide significance thresholds were set at alpha  = 0.05. The bayesint function was used to calculate the Bayes credible interval (95%) for estimation of the QTL location. The fitqtl function was used to calculate the percentage variance explained by the marker nearest the estimated QTL.

## Supporting Information

Figure S1 **Transition of Fish Velocity.** (A) Transition of maximum velocity during startle movement. HNI-II (n = 4), Hd-rR-II1 (n = 6), data from five days of trials. Bars represent SEM. In each strain, no significant effect of stimulus number was detected by one-way ANOVA (p>0.05). (B) Example of tracked velocity of one HNI-II individual through one trial.
(TIFF)

Figure S2 **Effect of Experimental Days on the Response Properties in the Four Inbred Strains.** HNI-II (n = 20), HNI-I (n = 6), HO5 (n = 14), Hd-rR-II1 (n = 20). Bars represent SEM. *** p<0.001 by two-way repeated measures ANOVA. No significant effect of days, and no significant interaction between day and strain were detected.
(TIFF)

Figure S3 **Whole Distribution of Values of the Response Properties in the Four Inbred Strains.** HNI-II (n = 20), HNI-I (n = 6), HO5 (n = 14), Hd-rR-II1 (n = 20). (A) Individual values of sensitivity in four inbred strains. (B) Individual values of response stability index in four inbred strains.
(TIFF)

Figure S4 **Startle Response Properties of F1 between HNI-II and Hd-rR-II1.** F1-a are the progeny of Hd-rR-II1 females and HNI-II males. F1-b are the progeny obtained from an opposite cross, between HNI-II females and Hd-rR-II1 males. HNI-II (n = 18), F1-a (n = 8), F1-b (n = 7) Hd-rR-II1 (n = 17) (A) Transition of response probability. Bars represent SEM. (B) Sensitivity of F1-a, F1-b, and their parental strains. Bars represent SEM. *p<0.05 and ** p<0.01 by Scheffe's F test. (C) Response stability index of F1-a, F1-b, and their parental strains. Bars represent SEM. a, b, and c indicate p<0.01 by Scheffe's F test.
(TIFF)

Figure S5 **Sexual Difference in the Response Properties in the Four Inbred Strains.** HNI-II (male, female  = 11, 9), HNI-I (male, female  = 3, 3), HO5 (male, female  = 6, 8), Hd-rR-II1 (male, female  = 10, 10). Bars represent SEM. ** p<0.01 and *** p<0.001 by two-way ANOVA. No significant effect of sex, and no significant interaction between sex and strain were detected.
(TIFF)

Figure S6 **Quantitative Trait Analysis for Sensitivity with F2 Obtained from Different Crosses.** In A and B, dashed lines indicate the thresholds (p = 0.05). (A) QTL analysis with F2 obtained from F1-a, the progeny of Hd-rR-II1 females and HNI-II males. QTL with a maximum LOD score of 4.42 located on linkage group 3 (explaining 20.9% of the variance) was detected (n = 29). (B) QTL analysis with F2 obtained from F1-b, the progeny of HNI-II females and Hd-rR-II1 males. No significant QTL was detected (n = 58).
(TIFF)

Movie S1 **Startle Response to Light-to-dark, not to Dark-to-light, Stimuli.** HNI-II fish are in the top-left and bottom-left, and Hd-rR-II1 fish are in the top-right and bottom-right. This movie is from a preliminary experiment, in which only visible light, not infrared light, was video recorded.
(MP4)

Movie S2 **Infrared Imaging under Entire Light-to-dark Background Changes.** The blinking of the small circle at the center of the image corresponds to that of the liquid crystal display placed above the fish. When the circle is brighter than the surrounding area, the liquid crystal display is brightly lit. When the circle is the same level of brightness as the surrounding area, the display has turned from light to dark. Note that the display practically covers the entire field, but the light-dark changes were recorded through a pinhole at the center of an infrared filter on a camera lens, which is why the change in lightning is shown only in a small circle.
(MP4)
