# Supplementary figures and images for: Genetic Control of Startle Behavior in Medaka Fish
Source: PLoS One. 2014 Nov 13;9(11):e112527. doi: 10.1371/journal.pone.0112527 (PMC4231031; doi:10.1371/journal.pone.0112527)

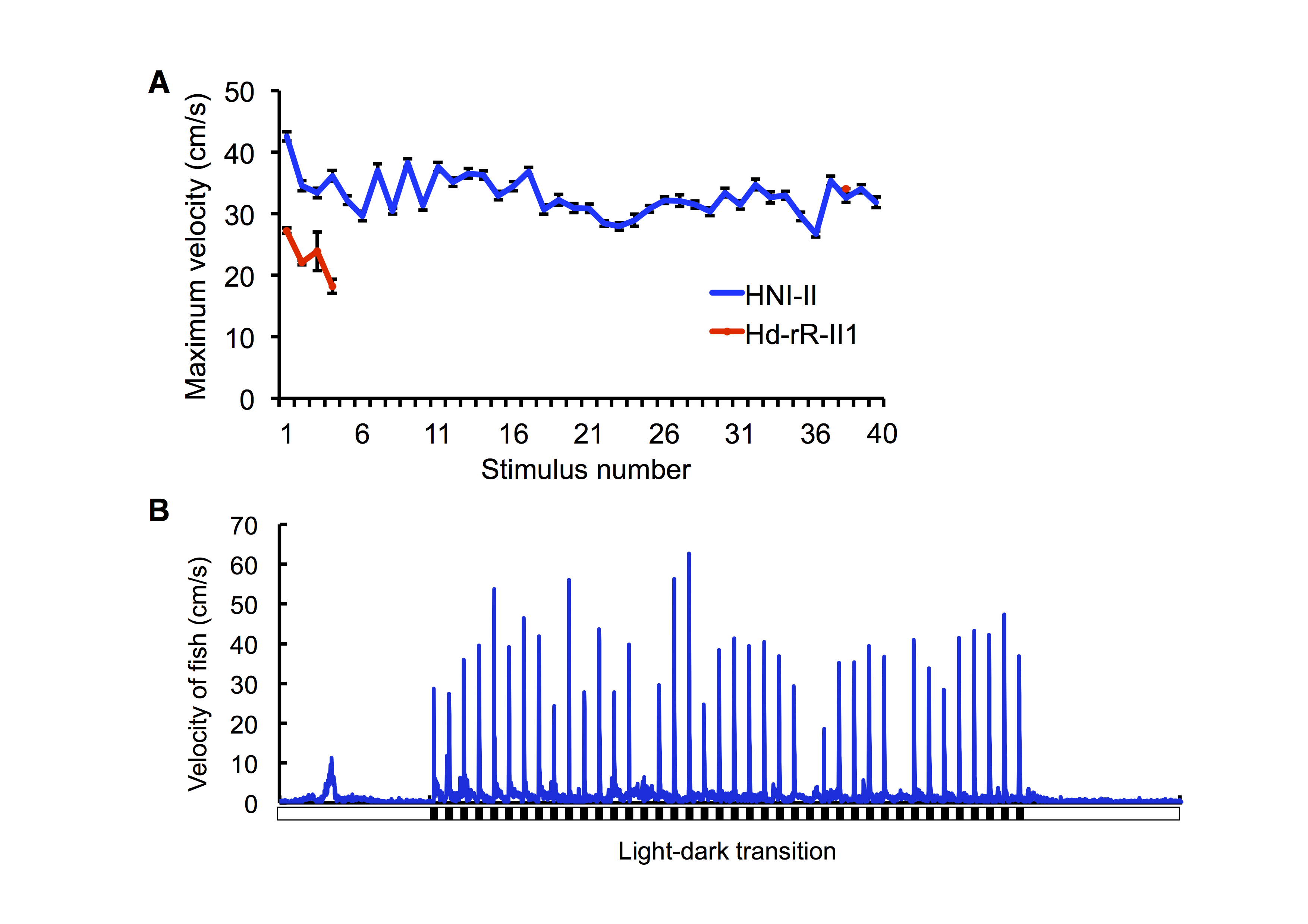

Supplement: Figure S1 — Transition of Fish Velocity. (A) Transition of maximum velocity during startle movement. HNI-II (n = 4), Hd-rR-II1 (n = 6), data from five days of trials. Bars represent SEM. In each strain, no significant effect of stimulus number was detected by one-way ANOVA (p>0.05). (B) Example of tracked velocity of one HNI-II individual through one trial. (TIFF) [file pone.0112527.s001.tiff]

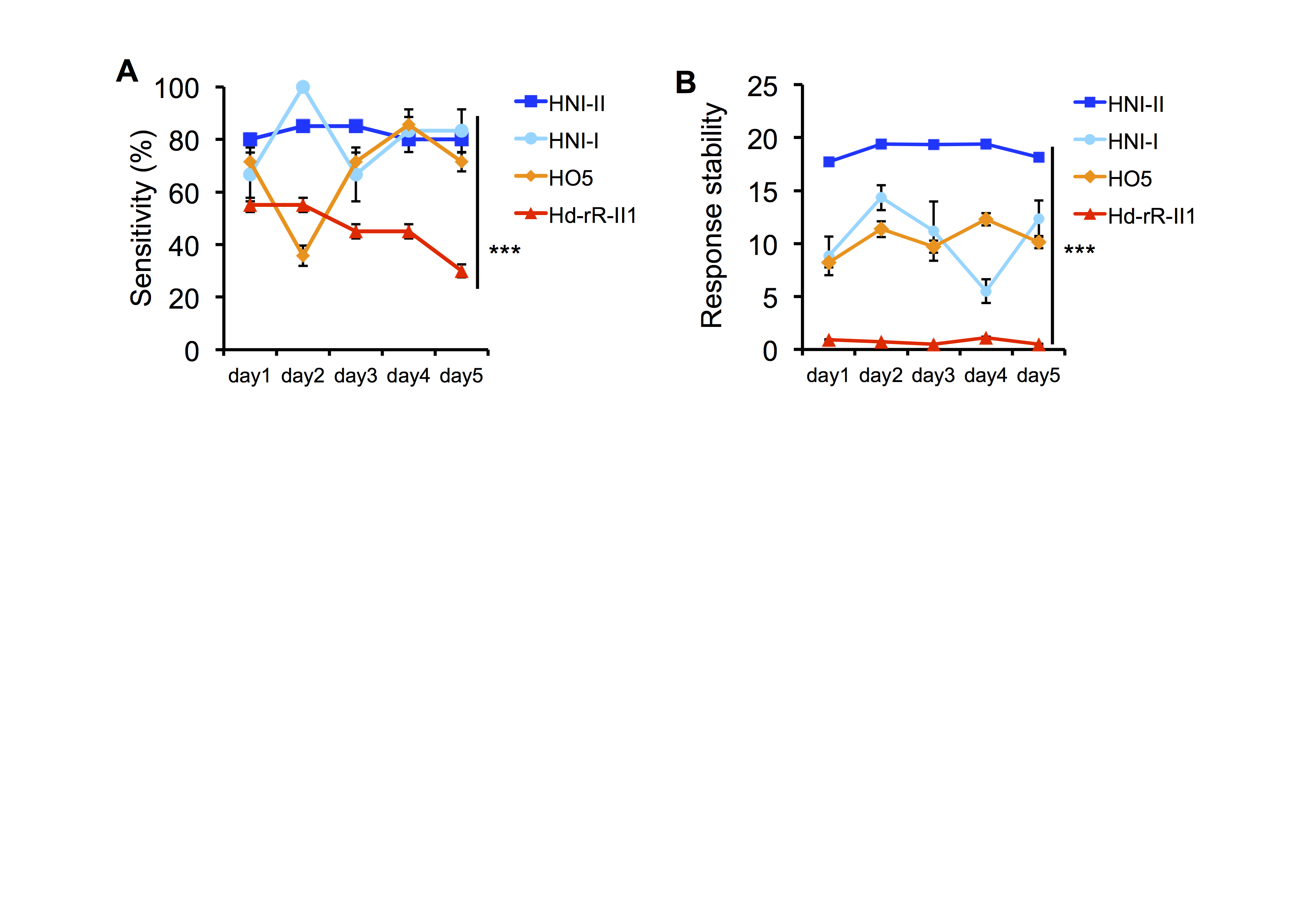

Supplement: Figure S2 — Effect of Experimental Days on the Response Properties in the Four Inbred Strains. HNI-II (n = 20), HNI-I (n = 6), HO5 (n = 14), Hd-rR-II1 (n = 20). Bars represent SEM. *** p<0.001 by two-way repeated measures ANOVA. No significant effect of days, and no significant interaction between day and strain were detected. (TIFF) [file pone.0112527.s002.tiff]

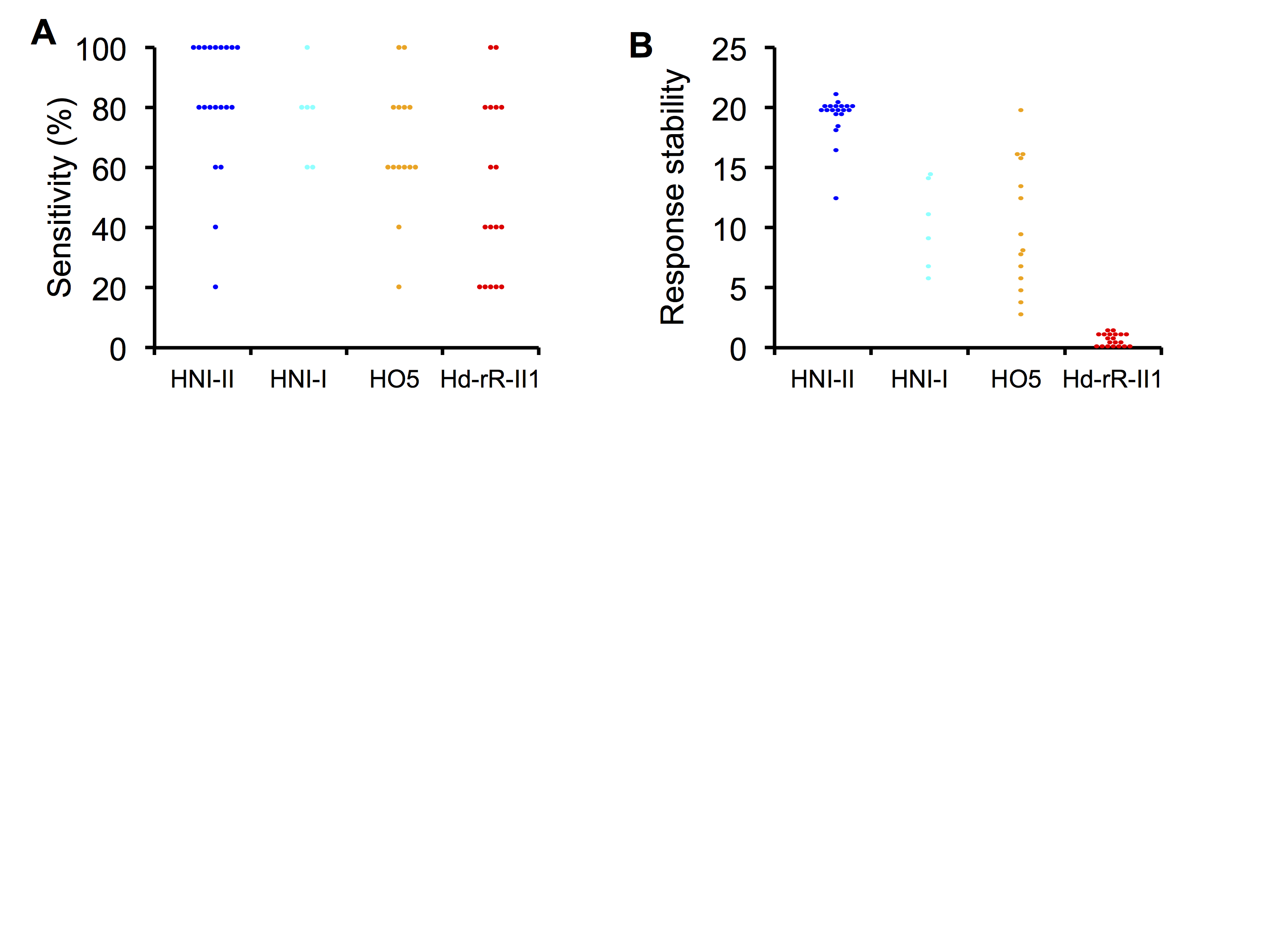

Supplement: Figure S3 — Whole Distribution of Values of the Response Properties in the Four Inbred Strains. HNI-II (n = 20), HNI-I (n = 6), HO5 (n = 14), Hd-rR-II1 (n = 20). (A) Individual values of sensitivity in four inbred strains. (B) Individual values of response stability index in four inbred strains. (TIFF) [file pone.0112527.s003.tiff]

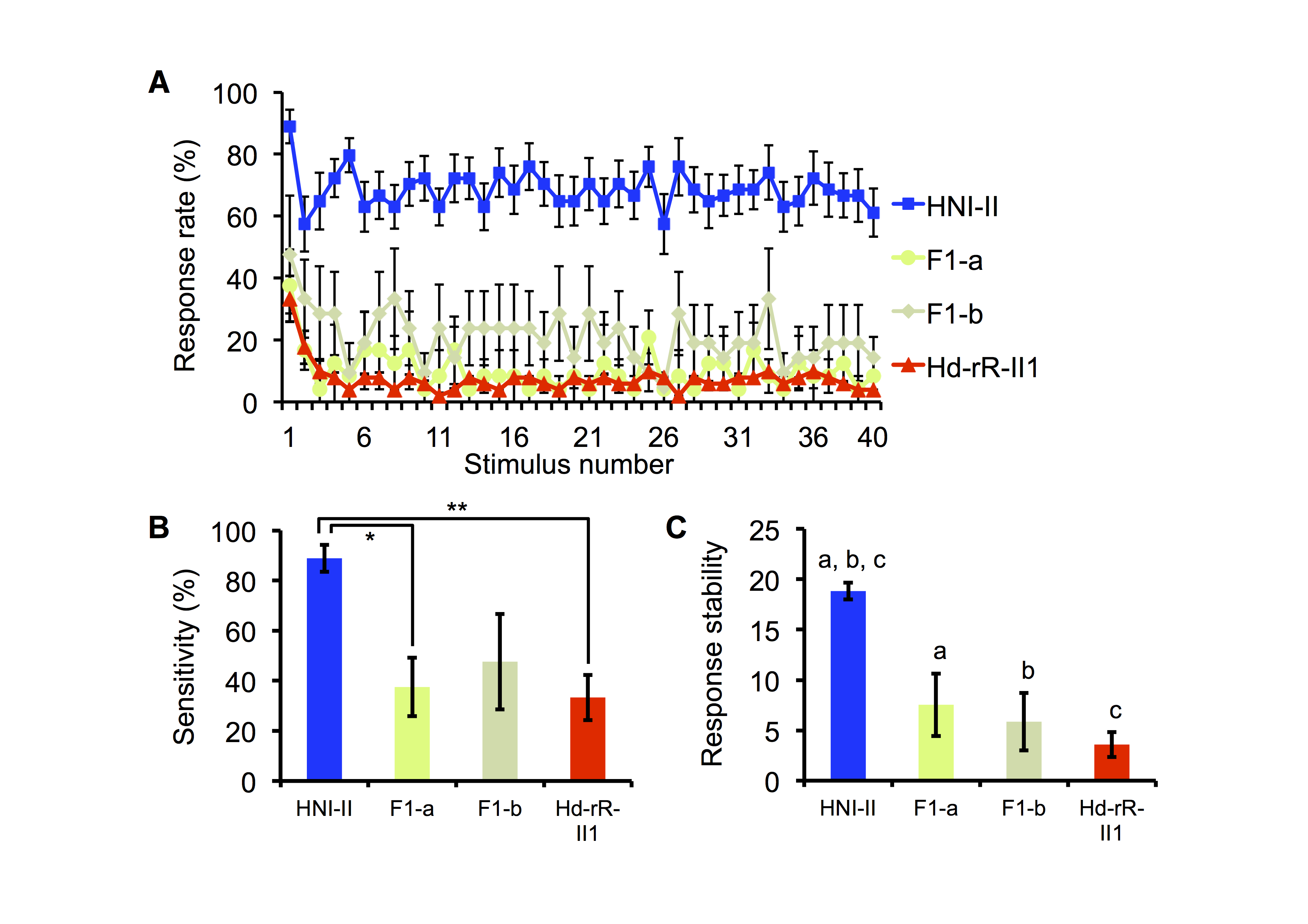

Supplement: Figure S4 — Startle Response Properties of F1 between HNI-II and Hd-rR-II1. F1-a are the progeny of Hd-rR-II1 females and HNI-II males. F1-b are the progeny obtained from an opposite cross, between HNI-II females and Hd-rR-II1 males. HNI-II (n = 18), F1-a (n = 8), F1-b (n = 7) Hd-rR-II1 (n = 17) (A) Transition of response probability. Bars represent SEM. (B) Sensitivity of F1-a, F1-b, and their parental strains. Bars represent SEM. *p<0.05 and ** p<0.01 by Scheffe's F test. (C) Response stability index of F1-a, F1-b, and their parental strains. Bars represent SEM. a, b, and c indicate p<0.01 by Scheffe's F test. (TIFF) [file pone.0112527.s004.tiff]

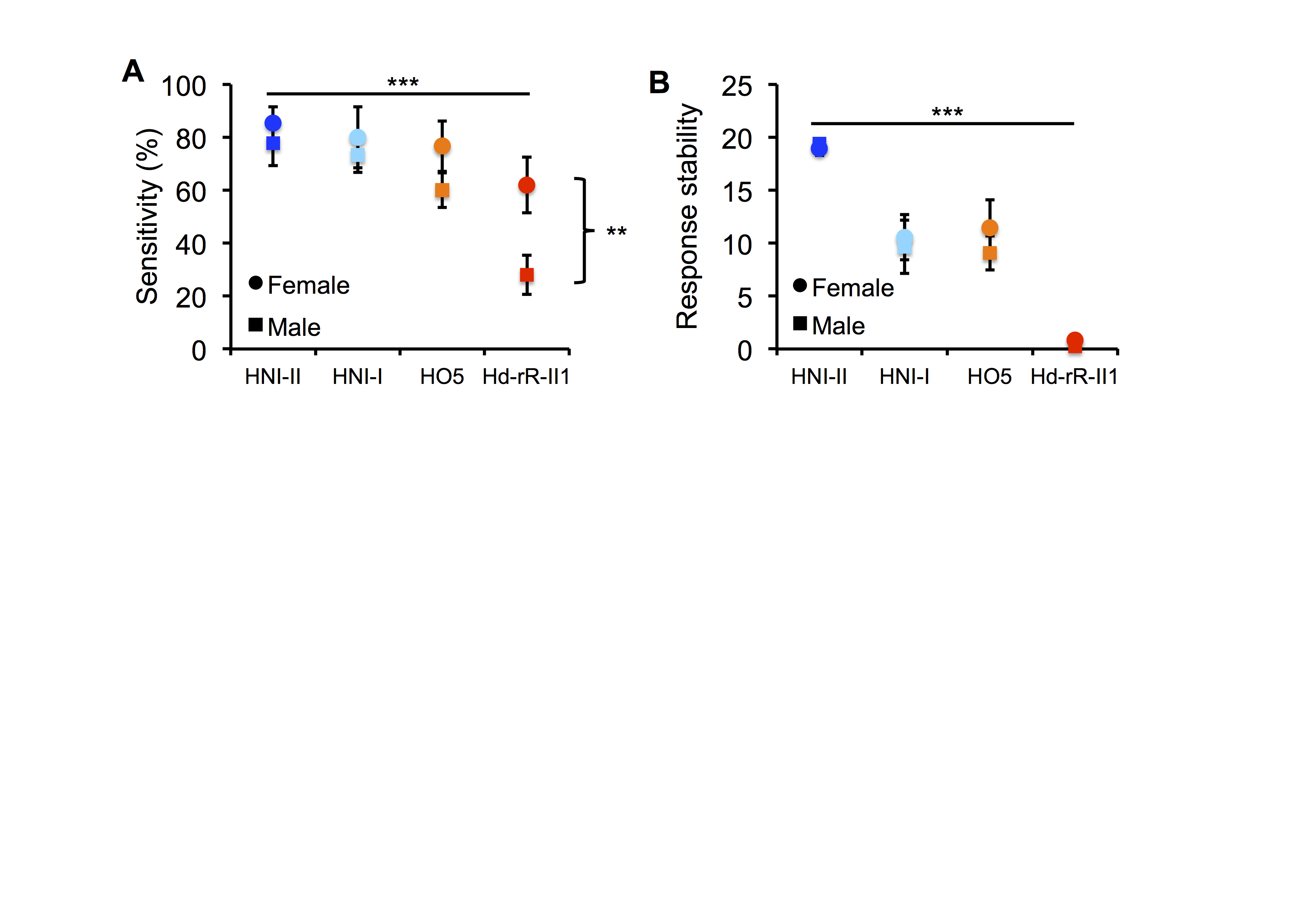

Supplement: Figure S5 — Sexual Difference in the Response Properties in the Four Inbred Strains. HNI-II (male, female = 11, 9), HNI-I (male, female = 3, 3), HO5 (male, female = 6, 8), Hd-rR-II1 (male, female = 10, 10). Bars represent SEM. ** p<0.01 and *** p<0.001 by two-way ANOVA. No significant effect of sex, and no significant interaction between sex and strain were detected. (TIFF) [file pone.0112527.s005.tiff]

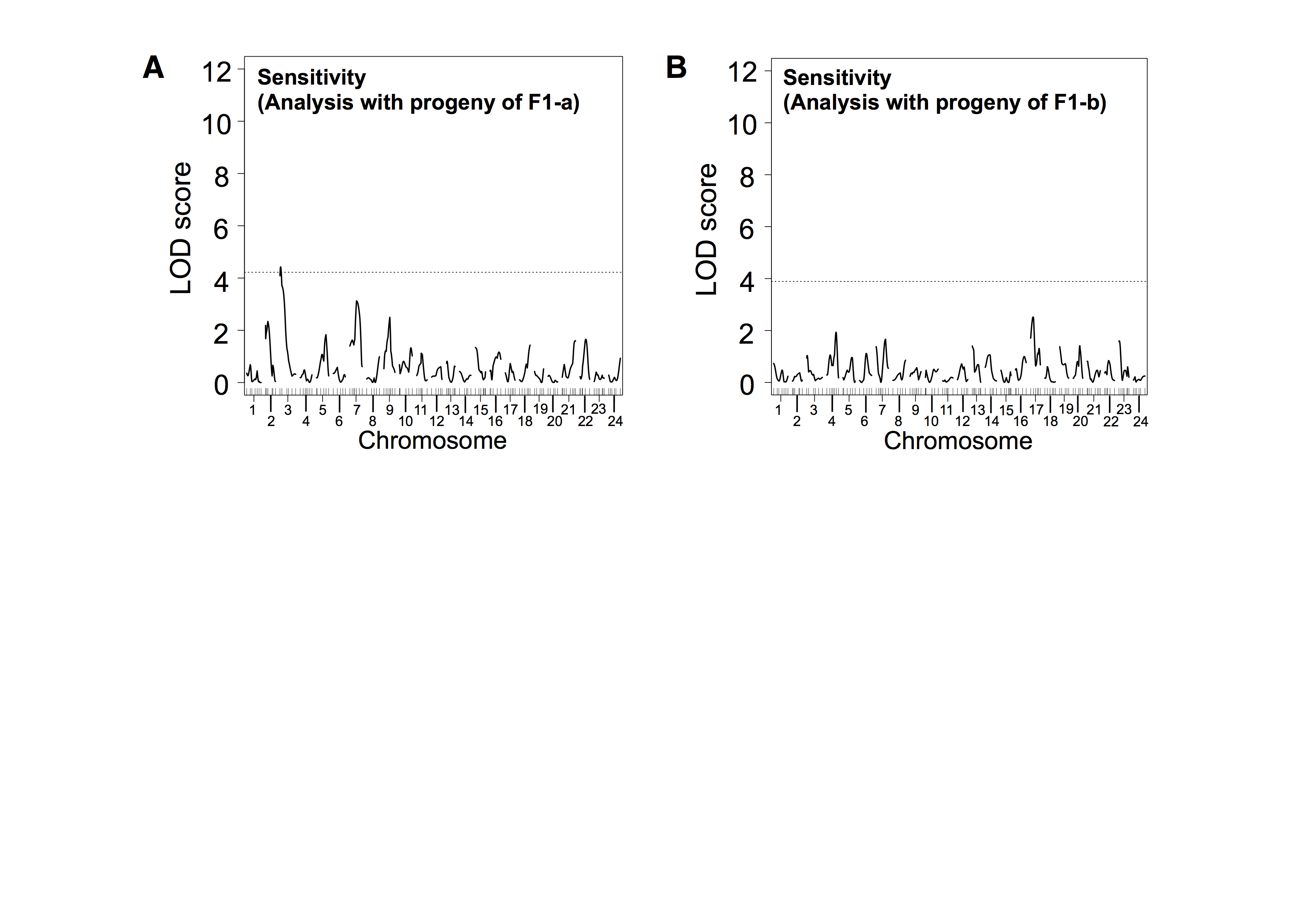

Supplement: Figure S6 — Quantitative Trait Analysis for Sensitivity with F2 Obtained from Different Crosses. In A and B, dashed lines indicate the thresholds (p = 0.05). (A) QTL analysis with F2 obtained from F1-a, the progeny of Hd-rR-II1 females and HNI-II males. QTL with a maximum LOD score of 4.42 located on linkage group 3 (explaining 20.9% of the variance) was detected (n = 29). (B) QTL analysis with F2 obtained from F1-b, the progeny of HNI-II females and Hd-rR-II1 males. No significant QTL was detected (n = 58). (TIFF) [file pone.0112527.s006.tiff]
